# Interplay between the gut microbiota and MAPK/NF-κ B/Nrf2 signaling in depression: pharmacological insights from traditional Chinese medicine

**DOI:** 10.3389/fphar.2026.1844902

**Published:** 2026-06-05

**Authors:** Jiale Yin, Jiaxuan Yin, Shijia Zhang, Xiaonan Xu, Xinran Wang, Zhaoxia Liu

**Affiliations:** 1 Heilongjiang University of Chinese Medicine, Harbin, Heilongjiang, China; 2 Guangzhou University of Chinese Medicine, Guangzhou, Guangdong, China; 3 First Affiliated Hospital of Heilongjiang University of Chinese Medicine, Harbin, Heilongjiang, China

**Keywords:** depression, gut microbiota, inflammatory signaling pathway, microbiota-gut-brain axis, TCM

## Abstract

Depression is a major global health burden. However, conventional pharmacotherapies often face limitations in efficacy and tolerability. In recent years, the relationship between the gut microbiota and depression has gradually emerged as a major research hotspot, offering new perspectives for the treatment of depression. The gut microbiota and the brain are connected through a bidirectional communication system known as the microbiota–gut–brain axis. Within this regulatory network, gut microbiota dysbiosis can disrupt intestinal barrier integrity, triggering systemic and central neuroinflammation. This neuroinflammatory cascade forms the critical pathological basis of depression. These pathological mechanisms underlying depression are driven by the dysregulation of key immune-inflammatory and oxidative stress signaling pathways, such as the mitogen-activated protein kinase (MAPK), nuclear factor-κB (NF-κB), and nuclear factor erythroid 2-related factor 2 (Nrf2) pathways. Therefore, targeting these pathways has become a promising strategy. Traditional Chinese Medicine (TCM), including botanical drug formulas and bioactive compounds, has demonstrated notable therapeutic efficacy. By altering the gut microbiota and strengthening the gut barrier, TCM can modulate the inflammatory signaling pathways mentioned above and decrease the levels of inflammatory cytokines such as tumor necrosis factor-alpha (TNF-α), interleukin-1beta (IL-1β), and interleukin-6 (IL-6). Consequently, inflammation in the central nervous system can be alleviated, excessive microglial activation can be inhibited, and depressive symptoms can be effectively improved. Owing to its multitarget and multicomponent characteristics, TCM has unique advantages in reshaping the gut microbiota; regulating key signaling pathways, such as MAPK, NF-κB, and Nrf2; and providing a promising therapeutic strategy for depression.

## 1 Introduction

Depression is a mental disorder with complex etiologies and severe clinical manifestations. Individuals with depression often experience persistent low mood or loss of pleasure or may exhibit abnormalities in at least five aspects, including appetite, sleep, attention, physical ability, self-worth, and suicidal tendencies (Trzeciak and Herbet, 2021; Wu et al., 2025). Depression has recently become a major global public health concern. Its high prevalence and chronic nature impose a substantial burden on public health systems and contribute to significant socioeconomic costs worldwide (McGrath et al., 2023).

Although the exact etiology of depression remains unclear, it is influenced by several factors, including changes in brain structure (Schmaal et al., 2016) and neuroendocrine systems (Gold, 2015). Traditional antidepressant therapies often encounter limitations, such as individual differences in drug tolerance and limited therapeutic efficacy (Yeung et al., 2018; Zhou et al., 2025). Moreover, severe side effects may occur, substantially reducing the quality of life of patients (Pillinger et al., 2023). Therefore, developing safer and more effective therapeutic approaches for depression is an urgent need.

Increasing evidence suggests that depression is closely related to inflammatory responses mediated by the gut microbiota. The gut microbiota can influence brain function through various pathways, including circulatory, immune, and neuroendocrine pathways (Dinan and Cryan, 2017). Patients with depression often exhibit gut microbiota dysbiosis, characterized by an increase in pathogenic bacteria and a decrease in beneficial bacteria. This imbalance can impair intestinal barrier integrity, allowing microbial antigens and proinflammatory factors to more easily enter the bloodstream, thereby activating inflammatory responses and oxidative stress (Maes et al., 2023; He et al., 2024). The activation of mitogen-activated protein kinase (MAPK) and nuclear factor-κB (NF-κB) signaling pathways, along with dysregulation of nuclear factor erythroid 2-related factor 2 (Nrf2) signaling, plays important pathological roles during this inflammatory process (Huang et al., 2025; Si et al., 2023). Interactions between gut microbiota dysbiosis and inflammatory responses can ultimately damage the central nervous system and promote the progression of depression (Yuan et al., 2023).

In light of these findings, modulation of the gut microbiota has emerged as a promising strategy for the treatment of depression. Although fecal microbiota transplantation (FMT) demonstrated therapeutic potential, a few patients still experience adverse effects, such as abdominal pain and constipation (Green et al., 2023). Similarly, probiotic treatments may lead to side effects, such as nausea and digestive problems, in some individuals (Nikolova et al., 2023). In recent years, botanical drug formulas and active metabolites of TCM have shown important effects in modulating the gut microbiota and alleviating depressive symptoms. Their unique advantages, including relatively few side effects and high patient acceptance, have been widely recognized in clinical treatment (Li et al., 2023; Bi et al., 2022).

Although both gut microbiota dysbiosis and inflammatory signaling are closely related to depression, systematic summaries of recent research progress remain limited. Therefore, this review focuses on immune-inflammatory mechanisms and discusses the relationship between the gut microbiota and depression. By restoring the balance of gut microecology and regulating inflammatory responses and metabolic signaling pathways, TCM may provide a unique and effective intervention strategy for the treatment of depression.

## 2 Methodology

We conducted a PubMed literature search for studies published between January 2016 and December 2025, with the language restricted to English. The following search strategy was used: (“Traditional Chinese Medicine” OR “TCM” OR “botanical drug” OR “Chinese botanical drug formulas”) AND (“depression” OR “depressive such as behavior”) AND (“gut microbiota” OR “intestinal bacteria”).

In the preliminary screening process, studies involving TCMs associated with the microbial–gut–brain axis and its active metabolites, including compounds such as saikosaponin A and ginsenoside, extracts such as *Semen Cuscutae* extract and *Gastrodia elata* Blume water extract, and formulas including Xiaoyaosan and Chaihu-shugan-san, were identified. We further identified the core targets associated with gut microbiota dysregulation and neuroinflammation, including MAPK, NF-κB, Nrf2, and NLRP3 signaling pathways. The search formula we end up with is: (“Saikosaponin A” OR “Ginsenoside” OR “Cuscutae semen” OR “Gastrodia elata” OR “Paeonia lactiflora” OR “Peach Gum” OR “Rosemary” OR “Gardeniae Fructus” OR “Xiaoyaosan” OR “Chaihu-shugan-san” OR “Shihosogansan” OR “Zhi-zi-chi” OR “Sini San” OR “Xiao-chai-hu-tang” OR “Fuzi-lizhong”) AND (“Depression” OR “depression” OR “depressive” OR “antidepressant”) AND (“Gastrointestinal Microbiome” OR “gut microbiota” OR “intestinal flora” OR “microbiome” OR “microbiota-gut-brain axis” OR “brain-gut axis” OR “Inflammation” OR “inflammatory” OR “neuroinflammation” OR “MAPK” OR “NF-kappa B” OR “NF-κB” OR “Nrf2” OR “NLRP3”).

The inclusion criteria for this study were as follows: clinical trials or *in vivo* animal studies investigating TCM interventions for depression; studies explicitly involving modulation of the gut microbiota and detailed investigation of at least one inflammatory signaling pathway, such as MAPK, NF-κB, Nrf2, or NLRP3; and studies with a control group in the experimental design. The exclusion criteria for this study were as follows: reviews, meta-analyses, conference abstracts, or case reports; studies lacking investigation of specific biological mechanisms; and articles for which the full text was unavailable.

A total of 202 relevant articles were initially retrieved. After screening based on the study design, data quality, and relevance of conclusions, studies that did not meet the inclusion criteria were excluded. Finally, 22 articles with high relevance for the topic were included in this review.

## 3 The gut microbiota in depression

The surface area of the human gastrointestinal tract is extremely large. This tract contains extremely complex and diverse microbial communities (Brown et al., 2022). Using a homemade microscope, Antonie van Leeuwenhoek first observed microorganisms in fecal samples, marking the discovery of gut microbes in human history (Kostka, 2025). According to current research, the human gastrointestinal tract contains between 10^13^ and 10^14^ microorganisms. In addition to archaea, fungi, and viruses, the gut microbiota is primarily dominated by bacterial phyla such as Firmicutes, Bacteroidota, Proteobacteria, and Actinobacteria (Cryan et al., 2019). Among these, Bacteroidota and Firmicutes are the most dominant phyla in the healthy human gut (Knuesel and Mohajeri, 2021). Nevertheless, the relative abundance and composition of these microbial communities are altered in patients with depression. Most studies have demonstrated that patients with depression exhibit relatively increased levels of Bacteroidota and decreased levels of Firmicutes (Yang et al., 2020; Liang S. et al., 2023), although some studies have reported contradictory findings regarding the abundance of Bacteroidota (Lin et al., 2017). Owing to substantial differences among research subjects and research methods, recent studies have increasingly focused on variations in the Firmicutes/Bacteroidota ratio in depression. In addition to alterations in Firmicutes and Bacteroidota, numerous studies have shown that proinflammatory bacteria such as *Eggerthella*, *Atopobium*, Enterobacteriaceae, and *Desulfovibrio* are more abundant in individuals with depression, whereas short-chain fatty acid (SCFA)-producing bacteria such as *Faecalibacterium* are less abundant (Knudsen et al., 2021; Simpson et al., 2021). Additionally, patients with depression had lower levels of bacteria such as *Bifidobacterium* and *Ruminococcus*. These bacteria are closely associated with the production of neurotransmitters such as γ-aminobutyric acid (GABA) and the synthesis of SCFAs. A reduction in these beneficial microbes may impair the body’s neuroprotective and anti-inflammatory processes (Cheung et al., 2019).

The pathophysiology of depression is intimately linked to gut microbiota dysbiosis. Pathogenic products may be translocated due to dysregulated gut microbiota, triggering a chain reaction of inflammatory responses extending from the peripheral system to the central nervous system. The microbiota–gut–brain axis is central to this process, while inflammatory signaling pathways such as MAPK, NF-κB, and Nrf2 also play important regulatory roles.

## 4 Gut microbiota and inflammatory signaling pathways in depression

### 4.1 MAPK signaling pathway

MAPKs are important intracellular signaling molecules and mainly include extracellular signal-regulated kinases (ERKs), Jun N-terminal kinases (JNKs), and p38 kinases (Albert-Gascó et al., 2020).

**FIGURE 1:**
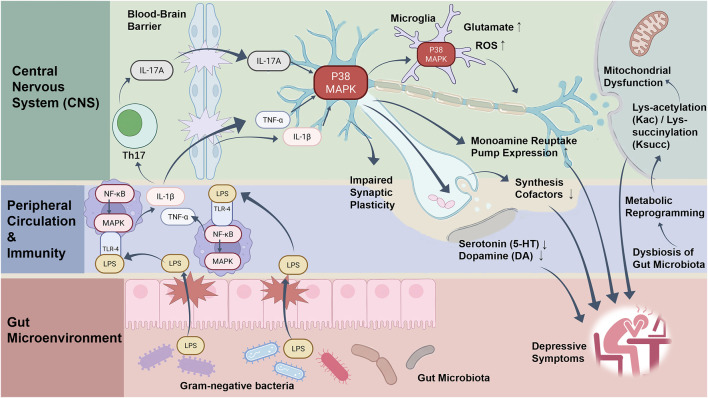
Schematic diagram of the mechanisms underlying depressive behaviors induced by gut dysbiosis *via* the MAPK signaling pathway. A disturbance in the gut microbiota may lead to an increase in the release of LPS. LPS triggers the TLR4/NF-κB/MAPK signaling cascade in peripheral immune cells, which stimulates Th17 cell differentiation and the release of the proinflammatory cytokines TNF-α and IL-1β. Peripheral proinflammatory factors and IL-17A cross the BBB and enter the brain parenchyma, where they activate the p38 MAPK pathway in neurons and microglia. Glutamate and ROS accumulate in microglia when p38 MAPK is activated. In neurons, p38 MAPK activation leads to the upregulation of monoamine neurotransmitter reuptake transporter expression, decreased concentrations of 5-HT and DA, and impaired synaptic plasticity. The gut microbiota also acts on abnormal Kac and Ksucc, leading to mitochondrial dysfunction. These metabolic pathological alterations and neuroinflammation work together to cause depressive symptoms.

The MAPK pathway can be activated by various stimuli, including stress and inflammation. This activation damages neurons and impairs hippocampal function by increasing the expression levels of several proinflammatory mediators and apoptotic signals (Meng et al., 2019). When gut microbiota homeostasis is disrupted, the levels of endotoxins such as lipopolysaccharides (LPS) become abnormally elevated. LPS is a potent inflammatory stimulus that influences glial cells and neurons in the central nervous system (CNS) by inducing immune cells to produce proinflammatory mediators (Dziedzic et al., 2024). Gut barrier dysfunction allows LPS derived from Gram-negative bacteria to activate the innate immune system through Toll-like receptor 4 (TLR4). The inflammatory responses and depressive behaviors in patients with major depressive disorder are closely linked with this immune activation (Caso et al., 2021) (Figure 1).

These proinflammatory signals exert their downstream effects mainly through p38 kinases. Cytokines such as IL-1β and TNF-α activate both the p38 MAPK and NF-κB signaling pathways, thereby disrupting the synthesis of neurotransmitters. Proinflammatory cytokines can significantly reduce serotonin (5-HT) and dopamine (DA) levels by increasing the expression of monoamine reuptake transporters and decreasing the availability of essential cofactors required for monoamine synthesis through the p38 MAPK pathway (Huang et al., 2025). Additionally, interleukin-17 A (IL-17 A) activates p38 MAPK signaling by binding to the IL-17RA receptor on hippocampal neurons (Hui et al., 2025). MAPK activation in microglia also promotes the production of reactive oxygen species (ROS) and glutamate release, which further damages the nervous system (Pinzi et al., 2025).

Gut microbiota dysbiosis can also alter hippocampal protein Lysine acetylation (Kac) and Lysine succinylation (Ksucc). These changes affect mitochondrial metabolism, synaptic function, and protein translation, thereby contributing to depressive behaviors and dysregulation of the MAPK signaling pathway (Liu et al., 2021).

### 4.2 NF-κB signaling pathway

**FIGURE 2:**
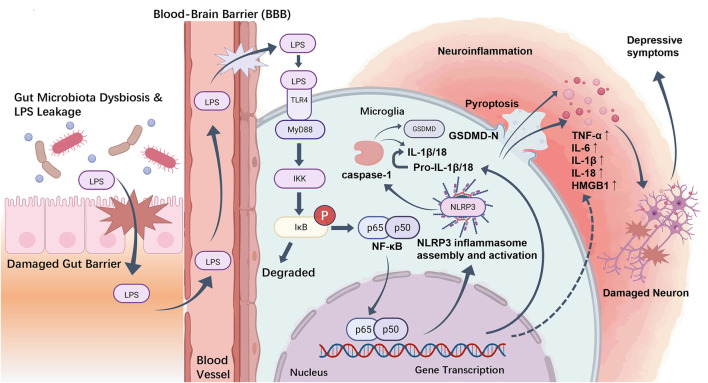
Schematic diagram of the mechanism by which gut microbiota dysbiosis induces depression through the NF-κB/NLRP3 signaling pathway. Disorder of the gut microbiota and damage to the gut barrier result in the leakage of bacterial-derived lipopolysaccharide (LPS) into the bloodstream. LPS affects the central nervous system by activating the TLR4 receptor on microglial cell surfaces, phosphorylating and degrading IκB *via* the MyD88/IKK signaling axis, and promoting NF-κB nuclear translocation. NLRP3, Pro-IL-1β, and Pro-IL-18 are expressed at relatively high levels when NF-κB enters the cell nucleus and initiates gene transcription. Cell pyroptosis is then triggered by the assembly of the NLRP3 inflammasome and the activation of caspase-1. Pro-IL-1β and Pro-IL-18 are also cleaved into mature forms by caspase-1. The proinflammatory cytokines TNF-α, IL-6, IL-1β, IL-18, and HMGB1 are released in large quantities when cell pyroptosis breaks down. This damages neurons and initiates a chain reaction of neuroinflammation, which ultimately leads to depressive symptoms.

Excessive activation of the classical NF-κB signaling pathway is closely associated with neuroinflammation in depression (Xiao et al., 2022) (Figure 2).

Gut microbiota dysbiosis can impair intestinal barrier integrity. Under these conditions, LPS can stimulate afferent vagal nerve fibers to send inflammatory signals to the brain or enter the CNS through the damaged blood–brain barrier. In brain regions such as the hippocampus, LPS binds to TLR4 receptors on microglia (Cao et al., 2025). Activated TLR4 then recruits the adaptor protein myeloid differentiation primary response 88 (MyD88) to mediate NF-κB phosphorylation, which initiates inflammatory responses (Abdelzaher et al., 2021). By upregulating protein 53 (p53) and its downstream target DNA damage-inducible transcript 4 (DDIT4), chronic stress can activate the NF-κB signaling pathway, resulting in neuroinflammation and depression like behaviors (Chen et al., 2023; Zhang K. et al., 2025). In addition, pathogen infection (Liu et al., 2017) and endogenous metabolites (Zhang et al., 2020) can also activate the NF-κB pathway.

The molecular process of NF-κB activation is tightly regulated. In the resting state, NF-κB generally exists as a protein 65–protein 50 (p65–p50) heterodimer that is inactive and bound to the inhibitory protein IκB in the cytoplasm. When cells are stimulated, this heterodimer is released because IκB kinase (IKK) is activated, phosphorylated, and cleaved. After the heterodimer translocates to the nucleus, activated NF-κB binds to DNA and initiates the transcription of several proinflammatory genes (Wang et al., 2023; Song et al., 2025).

NF-κB activation-mediated massive production of proinflammatory mediators, such as IL-1β, IL-6, TNF-α, inducible nitric oxide synthase (iNOS), and cyclooxygenase 2 (COX2), leads to a chronic inflammatory state (Cao et al., 2025; Li et al., 2021). Activated glial cells also play critical roles in this inflammatory process. Activation of the TLR4/MyD88/NF-κB pathway induces microglial polarization (Cao et al., 2022), which intensifies inflammatory responses and damages neurons (Song et al., 2025). Moreover, NF-κB activation may induce astrocytes to transform into the “A1” phenotype, causing additional neuroinflammation (Cong et al., 2023).

NF-κB signaling is also involved in the activation of the NOD-like receptor protein-3 (NLRP3) inflammasome (Liang X. et al., 2023). The NLRP3 inflammasome activates caspase-1, which cleaves inactive pro-IL-1β and pro-IL-18 into their mature forms (He et al., 2016). These cytokines exert strong proinflammatory effects by activating surrounding immune cells and amplifying inflammatory responses (Wang H. et al., 2025). Furthermore, caspase-1 cleaves the protein gasdermin D (GSDMD), generating an N-terminal fragment of GSDMD that forms pores in the cell membrane. This process disrupts the integrity of the cell membrane, leading to cell lysis and pyroptosis (Liu et al., 2016). Pyroptotic cells cause further exacerbate immune-inflammatory responses through the release of cellular contents, such as mature IL-1β, IL-18, and high mobility group box 1 (HMGB1) (Chen et al., 2022).

### 4.3 Nrf2 signaling pathway

**FIGURE 3:**
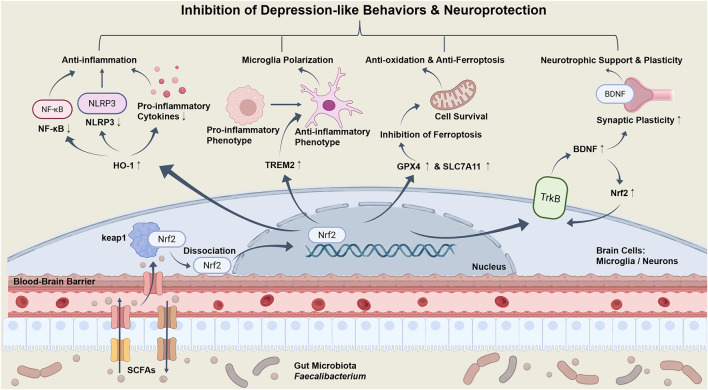
Schematic diagram of how the gut microbiota and metabolites alleviate depressive-like behaviors through the activation of brain Nrf2 signaling. SCFAs can be produced by the gut microbiota, including *Faecalibacterium*. After SCFAs cross the blood-brain barrier and enter the brain, they facilitate the dissociation and translocation of Nrf2 from the inhibitory protein Keap1 to the nucleus, which initiates the transcription of several genes that protect cells and are antioxidants. Nrf2 activation exerts neuroprotective effects primarily through the following pathways: activating HO-1, preventing the activation of NF-κB and NLRP3, and reducing the release of proinflammatory cytokines. Promoting the polarization of microglia from an inflammatory to an anti-inflammatory phenotype by upregulating TREM2 expression. Increasing the expression of GPX4 and SLC7A11 prevents ferroptosis and increases cell survival. Activating the BDNF/TrkB signaling axis within neurons enhances synaptic plasticity.

Nrf2 is a key transcription factor mainly involved in regulating anti-inflammatory and antioxidant reactions and preventing ferroptosis (Zuo et al., 2022). Nrf2 can inhibit the expression of inflammatory cytokines through the TLR4/NF-κB pathway (Huang et al., 2020). One important downstream target of Nrf2 is heme oxygenase 1 (HO-1), which degrades hemoglobin to generate carbon monoxide (CO) and bilirubin. These products inhibit NF-κB phosphorylation and prevent the release of proinflammatory factors (Zuo et al., 2022). Nrf2 can also compete with NF-κB for binding to the coactivator protein p300/CREB, thus preventing NF-κB-mediated neuroinflammation (Xie et al., 2023). Activation of the sirtuin-1 (SIRT1)/Nrf2 signaling pathway can also inhibit NLRP3 inflammasome activity in microglia (Arioz et al., 2019). Moreover, Nrf2 regulates microglial phenotypes. It induces the transition of microglia to an anti-inflammatory arginase 1+ phenotype by activating of the transcription of the triggering receptor expressed on myeloid cell-2 (TREM2) gene and exerting antidepressant-like effects (He et al., 2022). Thus, Nrf2 plays a crucial role in antidepressant mechanisms (Figure 3).

The pathophysiology of depression is closely associated with Nrf2 dysfunction. Both the Nrf2 expression level and activity are significantly reduced in a depression model (Si et al., 2023; Li et al., 2022). Nrf2 pathway inhibition reduces the expression of glutathione peroxidase 4 (GPX4) and solute carrier family 7 member 11 (SLC7A11) (Li et al., 2025; Zhang Y. et al., 2025), disrupting ferritin regulation and iron homeostasis in cells (Zeng et al., 2023). This impairment in antioxidant defense and enhancement of ferroptosis contribute significantly to the development of depression.

Nrf2 deficiency exacerbates neuroinflammation. Nrf2 knockout (KO) mice exhibit markedly elevated inflammatory responses, and their serum proinflammatory cytokine levels are considerably higher than those of normal mice (Yao et al., 2016). Furthermore, a bidirectional positive feedback loop exists between Nrf2 and the brain-derived neurotrophic factor (BDNF)- tropomyosin receptor kinase B (TrkB) signaling pathway. Nrf2 activation promotes BDNF expression, whereas BDNF signaling in turn activates Nrf2 (Sun et al., 2025; Alamri et al., 2023; Bruna et al., 2018). In depression, the ability of Nrf2 to activate the BDNF-TrkB pathway is impaired, resulting in neuronal atrophy and impaired synaptic plasticity due to the disruption of neurotrophic support (Chen T. et al., 2025; Ren et al., 2021).

Nrf2 signaling can also be regulated by the gut microbiota. Specific gut microbiota, such as *Faecalibacterium*, are major producers of SCFAs, which can regulate Nrf2 pathway activity. Through this mechanism, the gut microbiota can regulate the immune-inflammatory state, oxidative stress levels, and BDNF expression (Wen et al., 2025; González-Bosch et al., 2021).

### 4.4 Interaction between gut microbiota dysbiosis and the MAPK/NF-κB/Nrf2 signaling pathways

In conclusion, the pathophysiology of depression is significantly influenced by interactions between gut microbiota dysbiosis and the MAPK, NF-κB, and Nrf2 signaling pathways. Under dysbiotic conditions, increased Gram-negative bacteria release large amounts of LPS, which binds to TLR4 receptors on immune cells, activating NF-κB transcription, and simultaneously releasing proinflammatory mediators such as IL-1β, IL-6, and TNF-α. These overexpressed proinflammatory factors not only activate the downstream MAPK inflammatory signaling but also form a positive feedback loop to further amplify NF-κB activation and trigger more intense inflammatory responses (Fitzgerald and Kagan, 2020; Ciesielska et al., 2021).

As an antioxidant regulator, Nrf2 combines with HO-1 to protect cells from oxidative damage by blocking the excessive activation of MAPK and NF-κB signaling pathways and reducing inflammatory responses (Lim et al., 2020). However, Nrf2 expression is significantly reduced in depression, leading to severely impaired anti-inflammatory and antioxidant functions in nerve cells. This dysfunction represents an important mechanism underlying neuroinflammation in patients with depression.

## 5 TCM targets the gut microbiota and inflammatory pathways to treat depression

**FIGURE 4:**
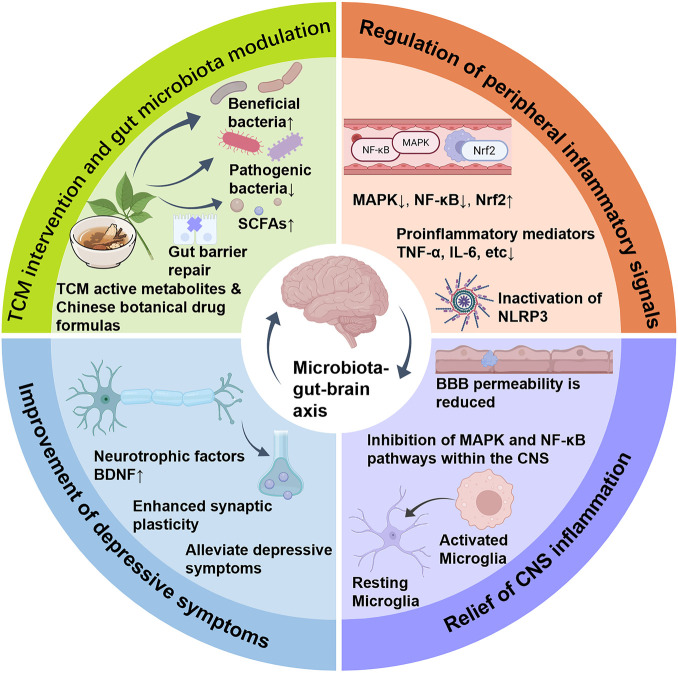
Schematic diagram of TCM targeting the gut microbiota and inflammatory pathways in the treatment of depression.

Figure 4 shows a schematic diagram that shows how traditional Chinese medicine can be used to target the gut microbiota and inflammatory pathways to treat depression.

### 5.1 Traditional Chinese medicine extracts and active metabolites

Table 1 summarizes how individual TCMs and their extracts target the MAPK, NF-κB, and Nrf2 signaling pathways to alleviate depression by regulating the gut microbiota. Detailed information on TCM metabolites and extracts is presented in Table 2.

**TABLE 1:** Summary of the mechanisms by which TCM metabolites and extracts regulate the gut microbiota and inflammatory signaling pathways.

TCM metabolites/Extracts	Model type/Pathological condition	Enriched microbiota (↑)	Depleted microbiota (↓)	Targets	Regulated signaling pathways	References
Saikosaponin A	RSP	Bacteroidota, *Akkermansia muciniphila*	Firmicutes	5-HT, DA, IL-1β, TNF-α, ROS, LPO, TNFR, NF-κB	TNFR1/NF-κB↓	Wang et al. (2025b)
Ginsenoside Rd	*E. coli* K1-induced anxiety/depression	*Oridobacter*	Enterobacteriaceae	BDNF, CREB, NF-κB, CORT, MPO	NF-kB↓	Han et al. (2020)
*Cuscuta chinensis* extracts	CUS	Firmicutes, *Lactobacillus*	Bacteroidota	NLRP3, NF-κB, COX-2, Nrf2, HO-1, IL-1β, TNF-α	NF-κB↓, NLRP3↓, COX-2↓; Nrf2↑	Hou et al. (2023)
*Gastrodia elata* Blume water extract/*Gastrodia elata* polysaccharides	CUMS/UCMS/sCSDS/LPS	Actinobacteriota, Bacteroidota, Lachnospiraceae, Ruminococcaceae, *Alloprevotella*, Defluviitaleaceae*_UCG-011*, *Bifidobacterium*, *Akkermansia*, *Bifidobacterium pseudolongum*	Firmicutes	MDA, SOD, BDNF, TrkB, Keap1, Nrf2, 5-HT, DA, SCFAs	Keap1-Nrf2/BDNF-TrkB↑	Wen et al. (2025), Huang et al. (2023), Huang et al. (2021)
*Paeonia lactiflora* polysaccharide	CUMS	Bacteroidetes	Proteobacteria, Actinobacteria	5-HT, IL-1β, IL-6, TNF-α, NLRP3, ASC, Caspase-1	NLRP3/ASC/Caspase-1↓	Zhou et al. (2023)
Peach Gum Polysaccharide	Orthotopic E0771 breast cancer	*Faecalibacterium*, *Roseburia*, *Bifidobacterium*	Lactobacillaceae	TLR4, NF-κB, NLRP3, Nrf2, ZO-1, Occludin	TLR4/NF-κB/NLRP3↓; Nrf2↑	Cui et al. (2025)
Rosemary extracts	CRS/LPS	Firmicutes, *Lactobacillus*	Bacteroidota, Proteobacteria	NF-κB, BDNF, IL-1β, TNF-α, IL-6	NF-κB↓	Guo et al. (2018)
Gardeniae Fructus oil	CUMS	*Muribaculaceae*, *Eubacterium_ventriosum group*	Lachnospiraceae*_NK4A136_group*, *Ruminococcus_torques_group*, *Acetitomaculum*	TLR4, NF-κB, NLRP3, 5-HT, DA, BDNF, IL-1β, IL-6, TNF-α, LPS, DAO	TLR4↓, NF-κB↓, NLRP3↓	Han et al. (2025)

Abbreviations: 5-HT, serotonin; CORT, corticosterone; CREB, cAMP-response element binding protein; CRS, chronic restraint stress; CUMS, chronic unpredictable mild stress; DA, dopamine; LPO, lipid peroxidation; MDA, malondialdehyde; MPO, myeloperoxidase; RSP, reserpine; sCSDS, subchronic and mild social-defeat stress; SOD, superoxide dismutase; UCMS, unpredictable chronic mild stress.

**TABLE 2:** Data extraction table of research on selected TCM metabolites/extracts.

TCM metabolites/Extracts	Type of extract	Research subjects	Control group (negative/Positive)	Tested dose/Concentration range	Minimal active concentration	Duration of intervention	References
Saikosaponin A (SSA)	SSA in NS	Male RSP mice	NC: NS (p.o.) PC: N/A	50 mg/kg/day	N/A	15 days	Wang et al. (2025b)
Ginsenoside Rd	Ginsenoside Rd in NS	*E. coli* K1-induced anxiety/depression mice	NC: NS (p.o.) PC: N/A	5 mg/kg/day	N/A	5 days	Han et al. (2020)
*Cuscuta chinensis* extracts	Ethanol extract	Male CUS mice	NC: NS (p.o.) PC: N/A	50, 100, 150 mg/kg/day	50 mg/kg/day	7 days	Hou et al. (2023)
*Gastrodia elata* Blume water extract (WGE)	Extract reconstituted in ddH2O	Male UCMS mice	NC: dH2O (p.o.) PC: Fluoxetine (20 mg/kg/day, p.o.)	WGE 5, 10, 20 mL/kg/day (eq. to 37.97, 75.94, 151.88 mg/kg gastrodin)	5 mL/kg/day	4 weeks	Huang et al. (2023)
*Gastrodia elata* Blume water extract (WGE)	Extract dissolved in ultrapure water	Male sCSDS mice	NC: pure water (p.o.) PC: N/A	500 mg/kg/day	N/A	30 days	Huang et al. (2021)
*Gastrodia elata* polysaccharides (GEP)	Purified GEP reconstituted in NS	Male CUMS mice, Male LPS mice	NC: NS (p.o.) PC: Fluoxetine (2.6 mg/kg/day, p.o.); St. John’s wort (150 mg/kg/day, p.o.)	50, 100, 200 mg/kg/day	100 mg/kg/day	28 days	Wen et al. (2025)
*Gastrodia elata* polysaccharides (GEP)	Purified GEP reconstituted in NS	LPS PC12 cell injury model	NC: Untreated PC12 cells PC: N/A	50, 100, 200, 500 μg/mL	100 μg/mL	24 h	Wen et al. (2025)
*Paeonia lactiflora* polysaccharide (PLP)	Purified PLP dissolved distilled water	Male CUMS mice	NC: distilled water (p.o.) PC: Fluoxetine (8 mg/kg/day, p.o.)	50, 100, 200 mg/kg/day	50 mg/kg/day	28 days	Zhou et al. (2023)
Peach Gum Polysaccharide	Polysaccharide freeze-dried powder dissolved in NS	Female tumor-bearing mice inoculated with E0771 breast cancer cells	NC: NS (p.o.) PC: N/A	400 mg/kg/day	N/A	10 days	Cui et al. (2025)
Rosemary extracts	RE dissolved in 1% Tween-80	Male CRS mice	NC: 1% Tween-80 (10 mL/kg, p.o.) PC: N/A	100 mg/kg/day	N/A	21 days	Guo et al. (2018)
Rosemary extracts	RE dissolved in phosphate buffer saline	LPS-stimulated BV-2 microglial inflammation model	NC: phosphate buffer saline treatment PC: N/A	5 µM	N/A	1h RE pretreatment, 3h LPS	Guo et al. (2018)
Gardeniae Fructus oil	Petroleum ether extract	Male CUMS mice	NC: Solvent (p.o.) PC: N/A	122 mg/kg/day	N/A	2 weeks	Han et al. (2025)

Abbreviations: CUMS, chronic unpredictable mild stress; CUS, chronic unpredictable stress; LPS, lipopolysaccharide; N/A, not applicable; NC, negative control; NS, normal saline; PC, positive control; p.o., *via* oral gavage; RSP, reserpine; sCSDS, subthreshold chronic social defeat stress; UCMS, unpredictable chronic mild stress.

Saikosaponin A (SSA) is a major metabolite of the TCM *Chaihu* (*Bupleurum chinense* DC.). Several studies have confirmed the antidepressant effects of SSA. In a reserpine-induced depression mouse model, SSA was administered *via* oral gavage at 50 mg/kg for 15 days. SSA effectively regulated the Firmicutes/Bacteroidota ratio in the gut microbiota and inhibited activation of the TNFR1/NF-κB signaling pathway (Wang M. et al., 2025). Another study using a chronic unpredictable mild stress (CUMS) rat model further clarified its pharmacologic mechanism. Continuous intragastric administration of SSA at 20 mg/kg for 6 weeks increased 5-HT and DA levels and inhibited the production of proinflammatory cytokines such as IL-1β, IL-6, and TNF-α. SSA also upregulated BDNF expression and inhibited TLR4/NF-κB signaling. Similar effects were observed *in vitro* in a PC12 cell model treated with corticosterone (CORT) (Tan et al., 2025).

Generally, the major active metabolites of *Renshen* (*Panax ginseng* C.A. Mey.) are called ginsenosides. They consist of various individual metabolites, such as Rd and Rb1, and have anti-inflammatory, antioxidant, and neuroprotective properties. Studies have demonstrated that Rd reduced the relative abundance of opportunistic pathogenic bacteria such as Enterobacteriaceae in *Escherichia coli* K1-induced depressive mice while increasing the relative abundance of beneficial bacteria such as *Odoribacter*, thereby restoring the balance of their gut microecology. Moreover, Rd reduced both peripheral and central inflammation responses and suppressed NF-κB expression (Han et al., 2020). Notably, Jiang et al. reported that administration of 35 mg/kg Rb1 to a chronic social defeat stress (CSDS) mice for 33 consecutive days increased BDNF signaling in the hippocampus and rescued neuronal damage in the hippocampus (Jiang et al., 2021). Liang et al. further demonstrated that the gut microbiota metabolized Rb1 into more pharmacologically active compounds, such as ginsenoside F2 (F2) and its metabolite K (CK). These metabolites promoted tryptophan metabolism, reduced inflammatory reactions in the hippocampus, and inhibited the MAPK/NF-κB signaling pathway (Liang et al., 2022). Moreover, Rb1 activated SIRT1 and Nrf2/HO-1 pathways, which significantly reduced NLRP3 inflammasome activation and alleviated oxidative stress and inflammation in the CNS (Jiang et al., 2022). Although the direct regulatory effects of Rb1 on the gut microbiota require further investigation, the critical role of gut microbiota in mediating the pharmacological processes of anti-inflammation and antidepression has become evident.

*Tusizi* (*Cuscuta chinensis* Lam.) is a botanical drug in TCM, and its extract contains various bioactive compounds such as flavonoids and polysaccharides. *Cuscuta chinensis* extracts improved gut microecology in chronic unpredictable stress (CUS) model mice by activating the expression of antioxidant genes, including Nrf2 and HO-1, while inhibiting the expression of proinflammatory signal molecules, such as NLRP3, NF-κB, and COX-2. These effects also suppress the excessive activation of microglia and astrocytes (Hou et al., 2023). Shao et al. reported that the major flavonoids in Semen Cuscutae flavonoids (SCFs) include hyperoside, quercetin, astragalin, and kaempferol. These flavonoids significantly reduced the abundance of Bacteroidota and *Bacteroides* in the gut of CUMS-induced depression model mice, while increasing the abundance of Firmicutes, *Lactobacillus,* and *Limosilactobacillus*. They also improved the metabolism of aldosterone, arachidonic acid, and bile acids, which are very important for preventing depression (Shao et al., 2025). Currently, direct experimental evidence for the regulatory effects of SCFs on MAPK, NF-κB, and Nrf2 signaling pathways is lacking. The precise mechanisms should be further explored in detail.

*Tianma* (*G. elata* Blume) is a well-known TCM. Its aqueous extract contains bioactive compounds such as gastrodin and 4-hydroxybutyl acrylate, which exert profound therapeutic effects against various mental disorders and emotional disorders. In depression-model mice, *G. elata* Blume water extract dramatically increased the abundance of beneficial bacteria, such as *Bifidobacterium* and greatly enhanced gut microecology, particularly among SCFA-producing bacterial groups, such as *Alloprevotella*, Ruminococcaceae, and Lachnospiraceae. Notably, within its classification, the *G. elata* Blume water extract increased the abundance of Lachnospiraceae and Ruminococcaceae but generally decreased the abundance of Firmicutes. These findings suggest that TCM greatly modulates the gut microecology during the course of treatment rather than indiscriminately eliminating bacteria (Huang et al., 2023; Huang et al., 2021). *Gastrodia elata* polysaccharides (GEPs) are the main active metabolites in the aqueous extract of *G. elata* Blume. They considerably increase the relative abundance of Bacteroidota in the gut of CUMS- and LPS- induced depression model mice. Moreover, Bacteroidota abundance was positively correlated with BDNF and TrkB levels and negatively correlated with Keap1 expression. These findings suggest that GEPs can regulate gut microbiota, suppress Keap1 expression, and increase BDNF-TrkB expression, which activates the Nrf2 pathway, decreases oxidative stress, and protects neurons (Wen et al., 2025).

*Baishao* (*Paeonia lactiflora* Pall.) derived from roots is mainly used in medicine. It is core botanical ingredient of Chinese botanical drug formulas such as Xiaoyaosan, which is used to “soothe the liver and relieve depression”. *Paeonia lactiflora* polysaccharide (PLP) is a key active metabolite of *Baishao*. PLP markedly reduces the abundance of Proteobacteria and Actinobacteria in the intestines of CUMS-induced depression model mice, while increasing the abundance of Bacteroidota. Similarly, it inhibits NLRP3 overactivation and the expression of proinflammatory factors, thereby exerting anti-inflammatory and antidepressant effects (Zhou et al., 2023). Studies have also demonstrated that NLRP3 inhibition can downregulate the expression of NF-κB pathway-associated proteins such as IKK, indicating that this process may inhibit NF-κB signal transduction (Mustafa et al., 2024). However, this hypothesis requires further experimental confirmation.

Peach gum polysaccharide, extracted from peach tree bark, increases the abundance of beneficial bacteria such as *Faecalibacterium*, *Roseburia*, and *Bifidobacterium* in the gut of model mice, activates AMPK and Nrf2 signaling pathways, and strengthens the gut mucosal barrier. Restoration of gut barrier integrity effectively prevents LPS from entering the bloodstream, inhibits the activation of the TLR4/NF-κB/NLRP3 inflammatory complex pathway, and promotes the production of other tryptophan metabolites and indole derivatives (Cui et al., 2025). In addition to improving gut microecology, rosemary extracts suppressed NF-κB expression in microglia and hippocampal cells (Guo et al., 2018). Similarly, *Gardeniae Fructus* oil regulated the gut microbiota in CUMS mice; downregulated inflammatory signaling molecules such as TLR4, NF-κB, and NLRP3; and exerted anti-inflammatory and antidepressant effects (Han et al., 2025).

Current evidence supporting the antidepressant effects of TCM metabolites is based on rodent models. However, these models fail to fully replicate the complex pathological and physiological processes of human depression. Moreover, the relationship between dosages used in animal studies and clinically equivalent dosages for humans remains unclear. Similarly, investigating crude botanical preparations such as *C. chinensis* extracts and *G. elata* Blume water extract presents distinct challenges, as their highly complex chemical compositions make the clear identification of the core active metabolites exceptionally difficult. Variations in the geographical origins and extraction methods of TCMs can cause variations in the proportions and contents of their metabolites, thereby directly affecting research outcomes. This variability may partly explain the considerable dosage differences reported among studies involving *G. elata* Blume water extracts. Furthermore, methods for establishing depression models and evaluation systems adopted by various studies are not uniform. This difference can be used as evidence to prove the therapeutic effects of TCM metabolite prescriptions on different pathological conditions, but it also causes poor comparability of research results. Despite the aforementioned limitations concerning methodologies and chemical compositions, the beneficial effects of these TCM metabolites in modulating the gut microbiota, regulating inflammatory pathways, and alleviating depressive symptoms remain evident.

In summary, active metabolites and extracts derived from TCMs, such as *Semen Cuscutae* extract, ginsenosides, and saikosaponin A, can reduce the number of opportunistic pathogens while increasing the abundance of beneficial bacteria. By strengthening the gut barrier and improving gut microecology, these compounds reduce systemic exposure to inflammatory mediators such as LPS. They further inhibit inflammatory signaling pathways in both peripheral and central systems, thereby significantly decreasing levels of proinflammatory cytokines such as IL-1β, IL-6, and TNF-α. Through regulation of the microbiota–gut–brain axis, this multitargeted and coordinated regulatory therapeutic strategy highlights the special advantages of active TCM metabolites in the treatment of depression.

### 5.2 Chinese botanical drug formulas

Table 3 illustrates how Chinese botanical drug formulas target MAPK, NF-κB, and Nrf2 signaling pathways to alleviate depression by modulating gut microbiota. Detailed information regarding Chinese botanical drug formulas is presented in Table 4.

**TABLE 3:** Summary of the mechanisms by which Chinese botanical drug formulas regulate the gut microbiota and inflammatory signaling pathways.

Chinese botanical drug formula	Model type/Pathological condition	Enriched microbiota (↑)	Depleted microbiota (↓)	Targets	Regulated signaling pathways	References
Xiaoyaosan	AIMD/PSD/CUMS	Firmicutes, Lachnospiraceae, *Faecalibaculum*, *Allobaculum*, *Monoglobus, Lactobacillus, Adlercreutzia*	Bacteroidetes, Bacteroidaceae, *Bacteroides, Ligullacoccus, Streptococcus, Staphylococcus, Corynebacterium*	P2X7R, TLR4, NLRP3, NF-κB, SCFAs, LPS, TNF-α, IL-1β, IL-6	P2X7R/NLRP3↓, TLR4/NLRP3↓, NF-κB↓	Hao et al. (2021), Chen et al. (2025b), Liu et al. (2024)
Chaihu-Shugan-San	CUMS/RS	Lactobacillaceae, Prevotellaceae, *Parabacteroides distasonis*	γ-Proteobacteria, Desulfovibrionaceae	BDNF, TrkB, NF-κB, TNF-α, IL-6, CORT, FXR	NF-κB↓; BDNF-TrkB↑	Han et al. (2021), Ma et al. (2022)
Zhi-zi-chi decoction	CUMS/CORT + CRS	Firmicutes, *Candidatus* Saccharibacteria, *Barnesiella*, *Lachnospiracea_incertae_sedis*, *Lactobacillus rhamnosus*	*Streptococcus*	butyric acid, 5-HT, DA, BDNF, Keap1, Nrf2, MAPK, NF-κB, TNF-α, IL-1β, IL-6	MAPK↓, NF-κB↓; Keap1-Nrf2↑	Liu et al. (2022), He et al. (2025), Tian et al. (2024)
Sini San	CUMS	Firmicutes, Lactobacillaceae, Ruminococcaceae, Oscillospiraceae, *Ligilactobacillus*	Muribaculaceae, Prevotellaceae, *Alloprevotella*, *Romboutsia*	5-HT, SCFAs, Caspase-1, ASC, NLRP3, BDNF, TrkB, PI3K, AKT, IL-18, IL-1β, TNF-α, ZO-1, Occludin-1, Claudin-1, AKT1, SRC, ALB	NLRP3↓; BDNF/TrkB/PI3K/AKT↑	Zhu et al. (2025), Zeng et al. (2025)
Xiao-Chai-Hu-Tang	CRS-induced CRC/Cancer with comorbid depression	Mice: *Bifidobacterium* Patients: Lactobacillales, *Anaerostipes*, *Bifidobacterium*	Mice: *Parabacteroides*, *Blautia*, Ruminococcaceae *bacterium* Patients: Ruminococcaceae *bacterium*	TLR4, MyD88, NF-κB, IκBα, p65, Bcl-2, Bcl-xL, IL-6, TNF-α, IL-1β, INF-γ	TLR4/MyD88/NF-κB↓	Shao et al. (2021)
Fuzi-Lizhong pill	CRS	*Lactobacillus*, *Bifidobacterium*	*Candidatus Arthromitus, Desulfovibrio*	EGFR, STAT3, PIK3R1, AKT1, mTOR, MAPK1, BDNF, TrkB, CORT, IL-6, IL-1β, TNF-α	MAPK↓; BDNF-TrkB↑, PI3K/Akt/mTOR↑	Zhao et al. (2025)

Abbreviations: 5-HT, serotonin; AIMD, antibiotic-induced microbiome depletion; CORT, corticosterone; CRC, colorectal cancer; CRS, chronic restraint stress; CUMS, chronic unpredictable mild stress; DA, dopamine; FXR, farnesoid X receptor; P2X7R, purinergic ligand-gated ion 7 receptor; PI3K, phosphatidyl-inositol 3-kinase; PSD, Poststroke depression; RS, restraint stress; TrkB, tropomyosin-related kinase B.

**TABLE 4:** Data extraction table of research on selected Chinese botanical drug formulas.

Chinese botanical drug formula	Composition, proportion and part used	Quality control	Type of extract	Research subjects	Control group (negative/Positive)	Tested dose/Concentration range	Minimal active concentration	Duration of intervention	References
Xiaoyaosan	*B. chinense* (root), *P. lactiflora* (root), *A. sinensis* (root), *A. macrocephala*/*A. lancea* (rhizome), *W. cocos* (sclerotium), *G. glabra* (root and rhizome), *M. canadensis* (aerial part), *Z. officinale* (fresh rhizome) Ratio = 5:5:5:5:5:4:1:5	UPLC-Q-TOF/MS analysis	Granules	Male AIMD mice	NC: NS (p.o.) PC: Probiotics (p.o.)	0.658 g/kg/day	N/A	14 days	Hao et al. (2021)
Xiaoyaosan	*B. chinense* (root), *P. lactiflora* (root), *A. sinensis* (root), *A. macrocephala*/*A. lancea* (rhizome), *W. cocos* (sclerotium), *G. glabra* (root and rhizome), *M. canadensis* (aerial part), *Z. officinale* (fresh rhizome) Ratio = 5:5:5:5:5:4:1:5	UHPLC analysis	Granules	Male PSD mice	NC: Sterile water (p.o.) PC: Fluoxetine (20 mg/kg/day, p.o.)	0.329, 0.658 g/kg/day	0.329 g/kg/day	21 days	Chen et al. (2025b)
Xiaoyaosan	*B. chinense* (root), *A. sinensis* (root), *P. lactiflora* (root), *A. macrocephala* (rhizome), *W. cocos* (sclerotium), *G. glabra* (root and rhizome), *M. canadensis* (aerial part), *Z. officinale* (fresh rhizome) Ratio = 6:6:6:6:6:3:2:2	HPLC analysis	Lyophilized aqueous extract	Male CUMS mice	NC: distilled water (p.o.) PC: Paroxetine (2.1 mg/kg/day, p.o.)	23.1 g/kg/day (Actual dose: 6.39 g/kg/day)	N/A	4 weeks	Liu et al. (2024)
Chaihu-Shugan-San	*B. chinense* (root), *C. reticulata* (pericarp), *C. anthriscoides* (rhizome), *C. rotundus* (rhizome), *C.* × *aurantium* (immature fruit), *P. lactiflora* (root), *G. glabra* (root and rhizome) Ratio = 9:9:9:9:9:15:5	HPLC analysis	Aqueous extract	Male RS mice	NC: NS (p.o.) PC: Buspirone (1 mg/kg/day, i.p.)	0.5, 1.0, 4.0 g/kg/day	1.0 g/kg/day	5 days	Han et al. (2021)
Chaihu-Shugan-San	*B. chinense* (root), *C. reticulata* (pericarp), *C. rotundus* (rhizome), *C.* × *aurantium* (immature fruit), *C. anthriscoides* (rhizome), *P. lactiflora* (root), *G. glabra* (root and rhizome) Ratio = 4:4:3:3:3:3:1	HPLC analysis	Aqueous extract	Male CUMS mice	NC: NS (p.o.) PC: N/A	20 mg/kg/day	N/A	8 weeks	Ma et al. (2022)
Zhi-zi-chi decoction	*G. jasminoides* (fruit), *G.* max (fermented seed) Ratio = 2:1	Botanical authentication, standardized extraction, and preestablished fingerprints	Purified ethanolic extract powder	Male CUMS rats	NC: 0.5% CMC-Na (p.o.) PC: Fluoxetine (2.1 mg/kg/day, p.o.)	10 g/kg/day	N/A	4 weeks	Liu et al. (2022)
Zhi-zi-chi decoction	*G. jasminoides* (fruit), *G.* max (fermented seed) Ratio = 1:1	LC-IT-TOF-MS and HPLC analysis	Ethanolic extract filtrate	*E. faecalis*, *L. rhamnosus* and *L. animalis*	NC: Untreated (0 μg/mL) PC: N/A	0, 3.125, 6.25, 12.5, 25, 50, 100, 200 μg/mL	12.5 μg/mL	*E. faecalis*: 6 h, *L. animalis*: 12 h, *L. rhamnosus*: 18 h	He et al. (2025)
Zhi-zi-chi decoction	*G. jasminoides* (fruit), *G.* max (fermented seed) Ratio = 17: 48	LC-MS analysis	Decoction	Male CRS+ CORT mice	NC: NS (p.o.) PC: venlafaxine (20 mg/kg/day, p.o.)	6, 12, 18 g/kg/day	6 g/kg/day	3 weeks	Tian et al. (2024)
Sini San	*B. chinense* (root), *P. lactiflora* (root), *C.* × *aurantium f. aurantium* (young fruit), *G. glabra* (root and rhizome) Ratio = 1: 1: 1: 1	HPLC analysis	Decoction	Male CUMS rats	NC: NS (p.o.) PC: Fluoxetine (1.58 g/kg/day, p.o.)	3.16 g/kg/day	N/A	6 weeks	Zhu et al. (2025)
Sini San	*B. chinense* (root), *P. lactiflora* (root), *C.* × *aurantium f. aurantium* (young fruit), *G. glabra* (root and rhizome) Ratio = 1: 1: 1: 1	Purchased from authorized commercial sources	Decoction	Male CUMS rats	NC: NS (p.o.) PC: Escitalopram oxalate tablets (1.05 mg/kg/day, p.o.)	2.5, 5.0 g/kg/day	2.5 g/kg/day	21 days	Zeng et al. (2025)
Xiao-Chai-Hu-Tang	*B. chinense* (root), *P. ginseng* (root and rhizome), *G. glabra* (root and rhizome), *Z. officinale* (fresh rhizome), *S. baicalensis* (root), *Z. jujuba* (fruit), *P. ternata* (tuber) specific ratio not specified	UHPLC/Q-TOF/MS analysis	Granules	Male CRS + MC38 mice	NC: NS (p.o.) PC: Fluoxetine (2.6 mg/kg/day, p.o.)	10.27, 20.54 g/kg/day	10.27 g/kg/day	14 days	Shao et al. (2021)
Xiao-Chai-Hu-Tang	*B. chinense* (root), *P. ginseng* (root and rhizome), *G. glabra* (root and rhizome), *Z. officinale* (fresh rhizome), *S. baicalensis* (root), *Z. jujuba* (fruit), *P. ternata* (tuber) specific ratio not specified	UHPLC/Q-TOF/MS analysis	Granules	Cancer patients with depressive symptoms	NC: Oral placebo PC: N/A	19 g, twice daily	N/A	6 weeks	Shao et al. (2021)
Xiao-Chai-Hu-Tang	*B. chinense* (root), *P. ginseng* (root and rhizome), *G. glabra* (root and rhizome), *Z. officinale* (fresh rhizome), *S. baicalensis* (root), *Z. jujuba* (fruit), *P. ternata* (tuber) specific ratio not specified	UHPLC/Q-TOF/MS analysis	Granules	Human CRC cell lines HCT116 and LoVo	NC: 0 μg/mL XCHT PC: N/A	10–320 μg/mL	No significant effect	48 h	Shao et al. (2021)
Fuzi-Lizhong pill	*A. carmichaelii* (processed lateral root), *C. pilosula* (root), *A. macrocephala* (rhizome), *Z. officinale* (dried rhizome), *G. glabra* (root and rhizome) Ratio = 2:4:3:2:2	UHPLC-MS/MS and HPLC-DAD analysis	Decoction of honey pills	Male CRS mice	NC: NS (p.o.) PC: Fluoxetine (2 mg/kg/day, p.o.)	1.5, 4.5, 13.5 g/kg/day	1.5 g/kg/day	4 weeks	Zhao et al. (2025)

Abbreviations: AIMD, antibiotic-induced microbiome depletion; CMC-Na, carboxymethyl cellulose sodium salt; CORT, corticosterone; CRC, colorectal cancer; CRS, chronic restraint stress; CUMS, chronic unpredictable mild stress; HPLC, high-performance liquid chromatography; HPLC-DAD, high-performance liquid chromatography-diode array detector; i.p., *via* intraperitoneal injection; LC-IT-TOF-MS, liquid chromatography-ion trap-time of flight mass spectrometry; LC-MS, liquid chromatography-mass spectrometry; N/A, not applicable; NC, negative control; NS, normal saline; PC, positive control; PSD, post-stroke depression; p.o., *via* oral gavage; RS, restraint stress; UHPLC, ultra-high-performance liquid chromatography; UHPLC-MS/MS, ultra-high-performance liquid chromatography-tandem mass spectrometry; UHPLC/Q-TOF/MS, ultra-high-performance liquid chromatography-quadrupole time-of-flight mass spectrometry; UPLC-Q-TOF/MS, ultra-performance liquid chromatography-quadrupole time-of-flight mass spectrometry.

Xiaoyaosan consists of *Chaihu* (*B. chinense* DC.), *Baishao* (*P. lactiflora* Pall.), *Danggui* (*Angelica sinensis* (Oliv.) Diels), *Cangzhu* (*Atractylodes lancea* (Thunb.) DC.)/*Baizhu* (*Atractylodes macrocephala* Koidz.), *Fuling* (*Wolfiporia cocos* (F.A. Wolf) Ryvarden and Gilb.), *Gancao* (*Glycyrrhiza glabra* L.), *Bohe* (*Mentha canadensis* L.), and *Shengjiang* (*Zingiber officinale* Roscoe). As a classic formula used in TCM to treat emotional disorders, Xiaoyaosan increases the abundance of butyric acid-producing bacteria, such as Lachnospiraceae, and decreases the abundance of pathogenic bacteria, such as *Streptococcus* and *Staphylococcus,* in the gut of depression model mice. It also strengthens the intestinal barrier and reduces LPS translocation. Xiaoyaosan decreases the expression of purinergic ligand-gated ion channel 7 (P2X7) protein, inhibits the activation of the NLRP3 inflammasome, and reduces the levels of proinflammatory cytokines such as TNF-α, IL-1β, and IL-6 (Hao et al., 2021; Chen Y. et al., 2025). Liu et al. reported that Xiaoyaosan reduced the abundance of harmful bacteria such as *Bacteroides* in the gut of CUMS rats, inhibited the excessive activation of the TLR4/NLRP3 signaling pathway in the colon and brain, downregulated NF-κB expression, and improved gastrointestinal symptoms and depressive behaviors (Liu et al., 2024).

Chaihu-Shugan-San is another TCM formula used to soothe the liver and relieve depression. It consists of *Chaihu* (*B. chinense* DC.), *Chenpi* (*Citrus reticulata* Blanco)*, Xiangfu* (*Cyperus rotundus* L.), *Zhiqiao* (*Citrus* × *aurantium* L.), *Chuanxiong* (*Conioselinum anthriscoides* (H.Boissieu) Pimenov and Kljuykov), *Baishao* (*P. lactiflora* Pall.), and *Gancao* (*G. glabra* L.). This formula reduced the abundance of inflammatory bacteria such as γ-Proteobacteria in the gut of restraint stress (RS) mice while increasing the abundance of probiotic bacteria including Lactobacillaceae and Prevotellaceae. Consequently, it prevented the entry of proinflammatory mediators such as LPS into the bloodstream, inhibited NF-κB activation, and lowered the levels of inflammatory factors such as IL-6 (Han et al., 2021). Chaihu-Shugan-San also modulated the metabolism of bile acids, inhibited hippocampal FXR activation, and upregulated BDNF and TrkB expression, thereby exerting nerve-nourishing and antidepressant effects (Ma et al., 2022).

Zhi-zi-chi decoction is composed of *Zhizi* (*Gardenia jasminoides* J. Ellis) and *Dandouchi* (*Glycine* max (L.) Merr.) and is widely used to treat emotional disorders. It restored the reduced abundance of Firmicutes in CUMS rats and increased the abundance of butyrate-producing bacteria such as *Lachnospiracea_incertae_sedis*. Butyric acid has anti-inflammatory properties, increases the levels of neurotransmitters such as DA and 5-HT, repairs damaged neurons, and reduces central inflammation (Liu et al., 2022). *In vivo* studies have further demonstrated that Zhi-zi-chi decoction significantly increased the abundance of *Lactobacillus rhamnosus*. Metabolites produced by this bacterium can bind to Keap1 and promote Nrf2 dissociation, thereby activating the Nrf2 pathway and strengthening antioxidant defense mechanisms (He et al., 2025). Tian et al. reported that oleanolic acid, a major active metabolite of Zhi-zi-chi decoction, regulates the MAPK and NF-κB signaling pathways and significantly suppresses inflammatory responses. Furthermore, transplantation of fecal microbiota from mice treated with Zhi-zi-chi decoction markedly alleviated anxiety- and depression-like behaviors in recipient mice, confirming that gut microbiota modulation is central to the in therapeutic effects of this formula (Tian et al., 2024).

Sini San consists of *Chaihu* (*B. chinense* DC.), *Baishao* (*P. lactiflora* Pall.), *Zhishi* (*Citrus* × *aurantium* f. aurantium), and *Gancao* (*G. glabra* L.), and is traditionally used to regulate emotional disorders. Sini San reduced inflammatory bacteria, such as Prevotellaceae, in CUMS model rats while increasing SCFA-producing bacterial communities such as Ruminococcaceae and Oscillospiraceae. Furthermore, it regulated tryptophan metabolism, increased SCFA production, and alleviated neuroinflammation (Zhu et al., 2025). Zeng et al. reported that Sini San effectively improved gut barrier integrity in CUMS model rats; reduced the levels of inflammatory cytokines such as IL-18, IL-1β, and TNF-α; and inhibited the expression of NLRP3 inflammatory complex-related proteins such as Caspase-1 and ASC in the hippocampus. In addition, Sini San activated the BDNF/TrkB/PI3K/AKT/CREB signaling pathway, reversed the CUMS-induced reduction of BDNF and TrkB expression in the hippocampus, and exerted neuroprotective effects (Zeng et al., 2025).

Xiao-Chai-Hu-Tang consists of *Chaihu* (*B. chinense* DC.), *Renshen* (*P. ginseng* C.A.Mey.), *Gancao* (*G. glabra* L.), *Shengjiang* (*Z. officinale* Roscoe), *Huangqi* (*Scutellaria baicalensis* Georgi), *Dazao* (*Ziziphus jujuba* Mill.), and *Banxia* (*Pinellia ternata* (Thunb.) Makino). It is a classic TCM formula traditionally used for the treatment of “Shaoyang syndrome”. Xiao-Chai-Hu-Tang alleviates systemic inflammation in patients with cancer-associated depression by remodeling the gut microbiota, reducing the abundance of proinflammatory taxa such as Parabacteroides, and enriching beneficial bacteria such as *Bifidobacterium*. These microbiota changes effectively reduce the systemic translocation of LPS, thereby suppressing activation of the TLR4/NF-κB signaling axis. These effects were further validated in a murine xenograft model of CRS-induced colorectal cancer (Shao et al., 2021). Subsequent studies also demonstrated that Xiao-Chai-Hu-Tang regulates tryptophan metabolism and neurotransmitter levels, activates the BDNF/TrkB/CREB and PI3K/AKT signaling pathways, and alleviates neuroinflammation, and promotes neuronal repair (Feng et al., 2025).

The Fuzi-Lizhong pill is composed of *Fuzi* (*Aconitum carmichaelii* Debeaux), *Renshen* (*P. ginseng* C.A.Mey.), *Baizhu* (*A. macrocephala* Koidz.), *Ganjiang* (*Z. officinale* Roscoe), and *Gancao* (*G. glabra* L.). In CRS mouse models, this formula increased the abundance of beneficial bacteria such as *Lactobacillus* and *Bifidobacterium*, activated BDNF-TrkB and PI3K/Akt/mTOR signaling pathways, inhibited MAPK signaling, and reduced inflammation in both the peripheral and central nervous systems (Zhao et al., 2025).

Research on the application of Chinese botanical drug formulas for regulating gut microbiota and treating depression still faces many limitations. Similar to studies on individual metabolites, current evidence is heavily reliant on rodent models. Most findings are unverified clinically in humans except for a limited number of therapies, such as Xiao-Chai-Hu-Tang for cancer-associated depression. Moreover, even among studies investigating the same classical formula, substantial differences exist in proportions, formulations, and dosage regimens, suggesting the need for further standardization of these interventional protocols. This inherent heterogeneity substantially elevates the difficulty of conducting comparative analyses among different studies. Interestingly, PLP increases the abundance of Bacteroidetes, but the regulatory activity of Xiaoyaosan, which contains Shaoyao, tends to decrease. However, both ultimately downregulate NLRP3 expression and inhibit the release of proinflammatory cytokines such as TNF-α, IL-1β, and IL-6. This phenomenon may be related to the highly complex gut microecology and the overall synergistic effects of Chinese botanical drug formulas on multiple metabolites. Although the regulatory effects on specific microbial communities differ, these interventions ultimately exert comparable antidepressant effects. Alongside the scrutiny of the aforementioned evidence, the integrity of the primary literature data also merits careful consideration. For instance, some studies on Sini San described both high- and low-dose treatment groups but reported outcomes for only one dosage group. Although these studies were retained to retain the breadth of evidence, such inconsistencies should be interpreted cautiously during data analysis.

Xiaoyaosan and Chaihu-Shugan-San are classic TCM prescriptions widely used for regulating emotions, and their antidepressant mechanisms have been extensively investigated. Overall, in addition to the use of botanical drugs such as *Gancao*, which are used to harmonize various medicinal metabolites, the application of *Chaihu* is particularly widespread. This might be closely associated with the efficacy of the extracts mentioned earlier. Compared with single metabolites, Chinese botanical drug formulas contain more complex combinations of bioactive compounds. Therefore, more advanced detection techniques and experimental designs are needed to identify the active metabolites for regulating gut microbiota and alleviating inflammatory responses. In conclusion, various Chinese botanical drug formulas can repair the gut barrier, regulate metabolic products, alter the gut microbial community, and suppress inflammatory signals. They also play a major role in simultaneously improving depressive symptoms through multiple-targeted synergy.

## 6 Discussion

An imbalance in the microbiota–gut–brain axis is a key mechanism for the occurrence and development of depression, among which the gut microbiota plays a major role as the initiating factor. Patients with depression frequently experience gut microbiota dysbiosis, which is characterized by a decrease in the abundance of SCFA-producing beneficial bacteria such as *Faecalibacterium* and an increase in opportunistic pathogens such as *Eggerthella* and *Atopobium*, as well as Gram-negative bacteria that produce LPS. This imbalance can damage intestinal barrier integrity, allowing proinflammatory mediators such as LPS to enter the bloodstream. This activity inhibits protective signaling pathways such as Nrf2 and activates proinflammatory signaling pathways such as MAPK and NF-κB, leading to inflammatory and metabolic disturbances associated with depression.

In terms of multitarget synergy and overall regulation, TCM exhibits special therapeutic benefits in the treatment of depression, where both its active metabolites and Chinese botanical drug formulas play crucial roles by regulating the gut microecosystem. Active TCM metabolites, such as peach gum polysaccharide, can repair damaged intestinal mucosal barriers, effectively preventing systemic translocation of proinflammatory mediators such as LPS. Simultaneously, they regulate gut microbiota composition and upregulate tight junction proteins such as ZO-1 and occluding, thereby maintaining internal homeostasis. Chinese botanical drug formulas such as Xiaoyaosan and Chaihu-Shugan-San can modulate gut microbiota and inflammatory pathways, thereby exerting antidepressant effects. Many distinct bacterial groups can perform similar physiological functions because of the complexity of the microecosystem. Consequently, although some formulas used in TCM have similar drug compositions, the effects of the regulated gut microbiota differ. Furthermore, variations in experimental models, detection methods, and the intestinal absorption and metabolism of Chinese botanical drug formulas and active metabolites contribute to differences in TCM-reshaped gut microbiota. Nevertheless, these interventions consistently reduced the abundance of proinflammatory bacteria and increased the growth of beneficial bacteria in the gut. Building upon this ameliorated gut microenvironment, TCM further exerts anti-inflammatory and antidepressant effects by downregulating TLR4-mediated NF-κB transcription, inhibiting excessive activation of the MAPK signaling pathway, preventing the assembly of the NLRP3 inflammasome, or activating Nrf2 signaling to inhibit excessive microglial activation. The therapeutic effects of TCM on depression through gut microbiota-mediated regulation of the MAPK/NF-κB/Nrf2 signaling pathway are summarized in this article, along with research advancements in this field.

Although significant progress has been made in the treatment of depression through TCM-mediated gut microbiota regulation, existing studies are still associated with limitations. Specifically, most discussions remain confined to macroscopic changes at the phylum level, failing to precisely pinpoint the key bacterial communities responsible for specific metabolic functions at the family, genus, and species levels. Changes in the gut microbiota are frequently detected and assessed only as secondary observations, and most studies have focused on confirming the clinical effects of treatments. Although this approach revealed a correlation between the gut microbiota and therapeutic outcomes, it failed to reflect the specific role of these microbial shifts during treatment. In addition, translating preclinical findings between species presents a significant challenge that requires rigorous scrutiny. Existing animal models have extensively elucidated the pharmacological mechanism of TCM, but evaluating its clinical translational validity is crucial. While murine gut microbiota shares structural and functional similarities with that of humans, significant differences remain in terms of taxonomic composition at genus and species levels, as well as in metabolite profiles. Therefore, although preclinical models have demonstrated that TCM exerts a significant regulatory effect on the microbiota–gut–brain, these effects need more rigorous validation in the clinical population. Larger clinical trials are warranted to further validate these findings. Beyond the evaluation of clinical efficacy, the safety profile of TCM demands equal scrutiny. Moreover, TCM clearly offers the advantage of engaging multiple biological targets; however, the inherent complexity of these botanical components leaves their potential adverse effects and overall toxicity largely unclear. This safety concern extends to the realm of integrative treatments. Related research has used antidepressants, such as fluoxetine, as positive controls. However, the interactions between Chinese medicines and antidepressants have not been investigated. The lack of mechanistic clarity restricts the safe and standardized clinical application of integrated Chinese and Western medicine.

To overcome the current research bottlenecks and clarify future development directions, we share short-term, medium-term, and long-term outlooks. Short-term outlooks: Future studies should leverage network pharmacology, high-throughput screening, and multiomics approaches to extensively mine key microbiota or its metabolites at genus and species levels, thereby clarifying the specific bioactive metabolites of TCM responsible for therapeutic effects. Medium-term outlooks: Promotion of the clinical transformation of animal experimental results by conducting large-scale multicenter randomized controlled clinical trials (RCTs). Moreover, the assessment of the toxicity of TCM and its interaction with conventional antidepressants should be prioritized, and standardized guidelines for medication should be developed. Long-term outlooks: Depression varies greatly from person to person. The relationship between TCM syndrome type and gut microbiota characteristics must be established, thereby exploring a personalized TCM treatment plan.

## 7 Conclusion

Despite the tremendous potential exhibited by TCM formulas and their active metabolites in alleviating depression by modulating the microbiota–gut–brain axis and MAPK/NF-κB/Nrf2 inflammatory pathways, current research continues to face several critical bottlenecks. Existing evidence relies predominantly on correlational animal studies, lacking definitive causal verification and robust clinical translation. Furthermore, botanical drug–drug interactions between TCM interventions and conventional antidepressants, along with their potential toxicological profiles, remain inadequately explored. To bridge these gaps, future research must prioritize large-scale, multicenter RCTs to establish reliable clinical efficacy and rigorously evaluate long-term safety and drug interactions. Building upon this foundation, mechanistic investigations should integrate multiomics approaches to precisely identify key TCM metabolites and microbial strains driving these therapeutic effects. Ultimately, establishing a mapping relationship between TCM syndrome patterns and specific gut microbiome signatures will provide a solid, evidence-based foundation for personalized and safe antidepressant strategies.
